# Relationship between sleep duration and hearing loss among adults aged 20–69: exploration of the U–shaped curve pattern

**DOI:** 10.3389/fnins.2025.1621044

**Published:** 2025-09-08

**Authors:** Xiaorong Yang, Keke Ju, Ruikai Wu, Boxin Liu, Qingxia Zhao, Tingting Jiang

**Affiliations:** ^1^Department of Otolaryngology - Head and Neck Surgery, Shaanxi Provincial People’s Hospital, Xi’an, China; ^2^School of Public Health, Xinjiang Medical University, Urumqi, China; ^3^Reproductive Medicine Center, Department of Obstetrics and Gynecology, The Second Affiliated Hospital of Air Force Medical University, Xi’an, China; ^4^The Second Surgical Department of Shaanxi Provincial People’s Hospital, Xi’an, China

**Keywords:** cross-sectional prevalence study hearing loss, sleep duration, U–shaped relationship, NHANES, restricted cubic spline

## Abstract

**Objective:**

The National Health and Nutrition Examination Survey (NHANES) data were used to explore the relationship between sleep duration and hearing level among adults aged 20 to 69 years, aiming to verify the association between abnormal sleep duration and hearing loss (HL).

**Study design:**

Cross-sectional prevalence study.

**Setting:**

The study utilized data from the NHANES, a large-scale, population-based, cross-sectional survey conducted in the United States. The NHANES is carried out by the National Center for Health Statistics (NCHS) to assess the health and nutritional status of the civilian, non-institutionalized US population.

**Methods:**

This study was based on the NHANES data from 2015 to 2016 and 2017 to 2020. A complex multistage probability sampling method was used to select adults aged 20–69 years. After excluding individuals with missing data, 4,883 participants were finally included. Sleep duration was collected through questionnaires, and hearing measurements were conducted by professional staff in mobile examination centers, including PTA at low frequencies, speech frequencies, and high frequencies. Statistical analysis was performed using a weighted linear regression model, adjusting for confounding factors such as gender and age. The restricted cubic spline (RCS) method was used to explore the non-linear relationship between sleep duration and hearing threshold. All statistical analyses were completed in the R environment, and *p*-value < 0.05 was considered statistically significant.

**Results:**

There was a U–shaped curve relationship between sleep duration and hearing threshold (*p*-overall trend < 0.001, *p*-non-linearity < 0.001), with the critical turning point at 8 h of sleep. Before this turning point, increasing sleep duration had a protective effect on the hearing threshold; after the turning point, excessive sleep duration led to an increase in the hearing threshold. After stratification by gender and age, a U–shaped curve relationship between sleep duration and hearing threshold was still observed in men and the elderly (*p*-overall trend < 0.001, *p*-non-linearity < 0.001). However, no dose–response relationship between sleep duration and hearing threshold was observed in women, young adults, and middle-aged adults (*p*-overall trend = 0.295, *p*-non-linearity = 0.158; *p*-overall trend = 0.447, *p*-non-linearity = 0.315; *p*-overall trend = 0.156, *p*-non-linearity = 0.777).

**Conclusion:**

There is a U–shaped curve relationship between sleep duration and hearing threshold, with the turning point at 8 h. Both short and long sleep durations have an adverse effect on hearing, and this phenomenon is particularly significant in men and the elderly. Future research needs to increase the sample size and adopt a prospective longitudinal study design. Meanwhile, Mendelian randomization and basic experimental studies can help to explore the underlying mechanisms in depth. Developing corresponding preventive strategies may help to reduce the potential burden of HL.

## Background

Hearing loss (HL) refers to a condition where there are organic or functional abnormalities in the auditory conduction system, sensory system of the body, and the various levels of nerve centers that comprehensively analyze sounds in the ear, leading to a decrease in auditory sensitivity. It is a significant public health issue (Nieman and Oh, 2020). According to the statistical data in 2019, the number of people with HL globally accounts for approximately 20.3% of the world’s total population. It is estimated that by 2050, this proportion will continue to rise to 30.47% (Haile et al., 2021). The impact of HL is extensive and profound. It not only increases the risk of depression (Fu et al., 2022), cognitive decline (Slade et al., 2020), and dementia (Griffiths et al., 2020) but also imposes a huge socioeconomic burden. The annual treatment costs associated with it reach up to 750 billion US dollars (Wilson et al., 2019).

Sleep is a crucial process for maintaining human physiological functions and mental health. High-quality sleep contributes to memory consolidation, emotional regulation, and enhancement of the immune system function. On the contrary, sleep disorders can have various adverse effects on human health. Currently, the high incidence of sleep disorders has attracted wide attention and has become a significant issue. Sleep disorders are prevalent globally and show an upward trend. In China, relevant studies indicate that the detection rate of sleep disorders among the general population ranges from 15.1% to 22.3%, while the prevalence rate among the elderly is as high as 35.9% (Cao et al., 2017; Lu et al., 2019). Sleep disorders not only reduce the quality of life but also increase the risk of various diseases, such as cardiovascular diseases, metabolic syndrome, and depression (Jung et al., 2018; Lawrence et al., 2020; Baiduc et al., 2023).

Sleep disorders may be associated with HL through potential mechanisms such as causing energy metabolism disorders, interrupting cochlear blood flow, and increasing oxidative stress (Mastino et al., 2023). However, research on the relationship between sleep disorders and HL is still insufficient. Most studies focus on the association between obstructive sleep apnea and HL. Existing studies have shown that patients with obstructive sleep apnea perform poorly in high-frequency hearing tests (Wang et al., 2022).

Abnormal sleep duration is also a type of sleep disorder. Reports indicate that One-third of American adults report getting less sleep than the National Sleep Foundation’s recommended 7–9 h (Mireku and Rodriguez, 2021). Non-optimal sleep duration is associated with adverse health outcomes. Short sleep duration is linked to hypertension, cardiovascular diseases, obesity, and type II diabetes (Johnson et al., 2021; Antza et al., 2022), while long sleep duration is related to cardiovascular diseases, stroke, and multiple sclerosis (Song et al., 2016; Ai et al., 2021). The cochlea is an important part of the peripheral auditory system, and the sensory hair cells within it are particularly sensitive to reactive oxygen species (ROS) (Poirrier et al., 2010). ROS accumulate during wakefulness, and antioxidants remove the excess ROS during sleep. Inadequate sleep can impair the ROS clearance process (Vaccaro et al., 2020). This plausible mechanism suggests a possible relationship between sleep duration and HL.

This study conducted a cross-sectional study to explore the relationship between sleep duration and hearing level among adults aged 20 to 69 years. This study relied on the latest data from the National Health and Nutrition Examination Survey (NHANES) in the United States. These data were from two survey cycles, 2015–2016 and 2017–2020, and included the sleep duration data and hearing test data of adults during these two cycles. This study hopes to use the data from these two cycles to provide more evidence to support the hypothesis that adults with abnormal sleep duration have poorer hearing.

## Research design and population

### Study population

The subjects of this survey were sourced from a complex multi-stage probability sampling survey conducted by the National Health and Nutrition Examination Survey (NHANES). This survey was jointly carried out by the Centers for Disease Control and Prevention and the National Center for Health Statistics. It consisted of an extensive initial household interview followed by a physical examination in a mobile examination center equipped with special facilities.

During the participant interview, questions related to demographics, socioeconomic status, diet, and health were covered. The physical examination part included physiological measurements, laboratory tests, etc., and all laboratory tests were conducted by trained medical staff. To ensure the representativeness of the sample, the sample weights needed to be corrected during the analysis process. The results of this survey are highly scientific in determining the prevalence of major diseases and disease risk factors.

**FIGURE 1:**
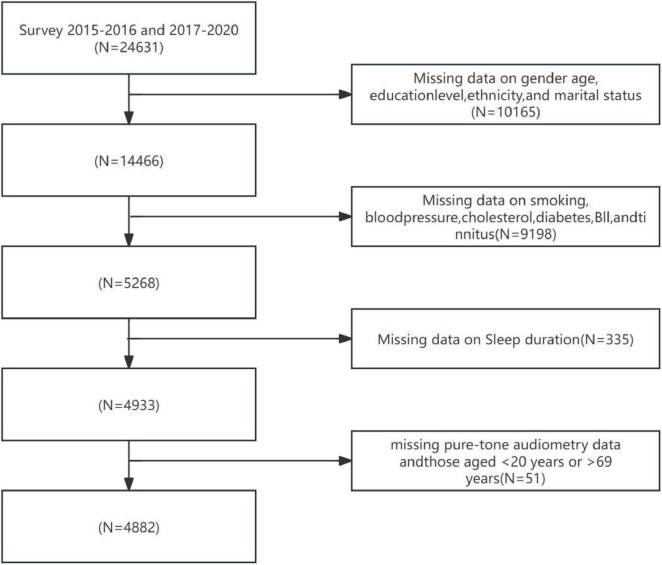
Flow chart.

Participants in this study were recruited from the 2015–2016 and 2017–2020 cycles of the NHANES. These cycles encompassed data on sleep duration and audiometric examination results for adults aged 20–69 years. Figure 1 illustrates the participant selection flowchart for this study. According to the standardized screening protocol depicted in Figure 1: Individuals with missing key demographic variables (sex, age, educational attainment, race/ethnicity, and marital status) were excluded, yielding a remaining sample of 14,466 participants; Individuals with missing health-related covariates (smoking history, blood pressure, total cholesterol, diabetes status, BMI, and tinnitus) were subsequently excluded, leaving 9,198 participants; Participants lacking sleep duration data were then excluded, resulting in 4,933 individuals; Finally, following the exclusion of individuals with missing pure-tone audiometry data and those aged < 20 years or > 69 years, a total of 4,882 participants with complete data on sleep (exposure), hearing (outcome), and covariates were included for statistical analysis. Participants were categorized into three age groups based on standard epidemiological stratification: Youth: 20–35 years, Middle-aged: 36–55 years, Elderly: 56–69 years.

### Sleep duration assessment

In this study, the sleep duration information was obtained from the NHANES Sleep Disorders Questionnaire, which is publicly available on the website.^1^ The question about sleep duration in the report was “How long do you usually sleep on weeknights?,” and the answers were rounded to the nearest half-hour. The short-sleep group was defined as those who self-reported sleeping less than 7 h (Xu et al., 2022). Despite inherent subjectivity, its validity as a standardized epidemiological instrument has been dually verified: (1) Objective calibration: Lauderdale et al. (2008) demonstrated significant moderate correlation with actigraphy-measured weekday sleep (OR = 0.47, *p*-value < 0.05); (2) Disease associations: Sleep metrics from this tool have revealed dose-response relationships with Stroke, metabolic syndrome, and all-cause mortality in leading journals (Hassani et al., 2024; Hu et al., 2024; Chen et al., 2025). Thus, despite individual-level error, this questionnaire reliably quantifies population-level sleep-health outcome associations, providing methodological foundation for investigating sleep-hearing loss mechanisms herein.

### Measurement of audiometry

Hearing measurements were conducted by trained examiners at the mobile testing center. The participants were aged between 20 and 69 years. Tympanometry was performed using a Titan tympanometer. Binaural pure-tone air-conduction audiometry was measured using an Interacoustics Model AD226 audiometer, TDH–49P headphones, and Etymotic EarTone 3A insert earphones. Participants who were unable to remove their hearing aids or could not tolerate the headphones were excluded. The entire measurement process is described in detail on the website.^2^

In this study, the low-frequency PTA was defined as the PTA value of the better ear at frequencies of 0.5, 1, and 2 kHz; the speech-frequency PTA was defined as the PTA value of the better ear at frequencies of 0.5, 1, 2, and 4 kHz; and the high-frequency PTA was defined as the PTA value of the better ear at frequencies of 4, 6, and 8 kHz. According to the guidelines of the World Health Organization (WHO), the severity of HL was defined based on the PTA value of the better-hearing ear at frequency points of 0.5, 1, 2, and 4 kHz (World Health Organization [WHO], 2024). The specific classifications are as follows: no HL: PTA value of 25 dB or lower; mild: 26–40 dB; moderate: 41–60 dB; severe: 61–80 dB; profound: 81 dB or worse (World Health Organization [WHO], 2024).

### Other variables

Potential covariates in the report include age, gender, race, education level, marital status, BMI, hypertension, diabetes, smoking, cholesterol, and tinnitus. This information was obtained through household interviews. Race: Non-Hispanic White, non-Hispanic Black, Mexican American, other Hispanic, other races (including multi-racial). Education level: Below high school, high school, above high school. Marital status: Married, unmarried. BMI: Calculated as weight divided by the square of height (kg/m^2^). Specifically, BMI < 18.5 is considered underweight, 18.5 ≤ BMI < 24 is considered normal, 24 ≤ BMI < 28 is considered overweight, 28 ≤ BMI < 34 is considered obese, and BMI ≥ 34 is considered extremely obese. Smoking status: Determined by asking “Have you smoked at least 100 cigarettes in your lifetime?” and “Are you currently a smoker?” Hypertension: Determined by asking “Has a doctor ever told you that you have high blood pressure?” Diabetes: Determined by asking “Has a doctor ever told you that you have diabetes?” Cholesterol status: Determined by asking “Has a doctor ever told you that you have high cholesterol?” Tinnitus: Determined by asking “In the past 12 months, have you been bothered by ringing, roaring, or buzzing in your ears that lasted for 5 min or longer?” Those who answered “yes” were considered to have a history of tinnitus.

### Statistical analysis

All analyses were conducted using R 4.3.1. Continuous data were presented as mean ± standard deviation, and categorical data were presented as percentages (*N*%). To make the sample representative of the non-institutionalized civilian population in the United States, the full–sample 2-year interview weights (WTINT2YR) for the 2015–2016 cycle and the interview weights (WTMECPRP) for the 2017–2020 cycle were used to adjust for oversampling, survey non-response, non-coverage, and post-stratification (Mirel et al., 2013; Chen et al., 2018).

Given that the NHANES survey adopted a complex stratified sampling design to ensure national representativeness, the means, standard errors, percentages, and *P*-values reported in this study were all weighted values. Count data were statistically described as percentages (%), and chi-square tests were used for inter-group comparisons of count data. A weighted linear regression model was used to evaluate the relationship between sleep duration and hearing threshold. In the linear regression model, sleep duration was regarded as the independent variable, and hearing threshold was regarded as the dependent variable. Meanwhile, to reduce the interference of other factors on the results, adjustments were made for gender, age, and other potential confounding factors. The above–mentioned weighting variables WTINT2YR and WTMECPRP were also used in the regression model. To explore the non-linear relationship between sleep duration and hearing threshold, the RCS method was employed in this study. The RCS method can capture the non-linear patterns between variables and generate smooth curves for each variable. Four knots were set to define the shape of the spline function, so as to better fit the non-linear trends in the data. Similar to the linear regression analysis, confounding factors such as gender and age were also included in the RCS analysis. All data processing and statistical analyses were completed in the R environment. The “survey” package was used for weighted analysis, and the “rms” package was used for restricted cubic spline analysis. A *P*-value less than 0.05 was considered statistically significant.

## Results

### Distribution of baseline data

Table 1 shows the distribution of basic characteristics between the case group (participants with hearing loss, HL) and the control group (non-HL participants). A total of 4,883 subjects were included in the study, with 1,066 (weighted percentage: 57.41%) assigned to the case group (HL group). In the case group, the weighted proportions of males, non-Hispanic Whites, and those with an education level above high school were 57.41%, 47.56%, and 58.71%, respectively. Compared with the control group, the case group had a higher proportion of males and non-Hispanic Whites, a lower proportion of those with an education level above high school and married individuals, and more common situations of being unmarried, smoking, having diseases such as hypertension, and suffering from tinnitus. The proportion of the elderly population was larger, and the proportions of extremely obese and obese individuals were higher. The *p*-values of all the above comparisons were less than 0.001. The proportion of those with short sleep duration was slightly higher, but the difference was not statistically significant (*p*-value > 0.05).

**TABLE 1:** Comparison of basic characteristics between HL group and non-HL group.

Variable	N		Unadjusted weights			Weight		
	Case group	Control group	Case group (%)	Control group (%)	*p*-value	Case group (%)	Control group (%)	*p*-value
Gender (%)					0.001			
Male	612	1,730	53.99	46.66		57.41	46.06	< 0.001
Female	454	2,026	46.01	53.34		42.59	53.94	
Race (%)					< 0.001			< 0.001
Non-Hispanic White	507	1,231	74.13	64.46		47.56	32.77	
Non-Hispanic Black	168	869	6.94	11.68		15.76	23.14	
Mexican American	149	622	5.65	8.78		13.98	16.56	
Other Hispanic	127	464	5.3	6.4		11.91	12.35	
Other races- including multi-racial	115	570	7.97	8.68		10.79	15.18	
Educational (%)					< 0.001			< 0.001
High school	256	847	24.05	21.03		24.02	22.55	
Above high school	501	2,205	58.24	67.28		47	58.71	
Below high school	309	704	17.71	11.69		28.99	18.74	
Marital status (%)					0.036			< 0.001
Unmarried married	458	1,851	39.21	82.24		42.96	49.28	
Unmarried married	608	1,905	60.79	78.79		57.04	50.72	
Smoking (%)					< 0.001			< 0.001
No	491	2,258	47.49	58.07		46.06	60.12	
Yes	575	1,498	52.51	41.93		53.94	39.88	
Hypertension (%)					< 0.001			< 0.001
No	424	2,559	42.34	71.51		39.77	68.13	
Yes	642	1,197	57.66	28.49		60.23	31.87	
High cholesterol (%)					< 0.001			< 0.001
No	470	2,551	42.32	68.66		44.09	67.92	
Yes	596	1,205	57.68	31.34		55.91	32.08	
Diabetes (%)					< 0.001			< 0.001
No	752	3,307	75.06	91.07		70.54	88.05	
Yes	314	449	24.94	8.93		29.46	11.95	
Tinnitus (%)					< 0.001			< 0.001
No	763	3,272	68.17	85.82		71.58	87.11	
Yes	303	484	31.83	14.18		28.42	12.89	
BMI (%)					< 0.001			< 0.001
Severely obese	238	841	24.46	20.71		22.33	22.39	
Obese	389	1,152	37.84	30.39		36.49	30.67	
Underweight	10	50	1.14	1.3		0.94	1.33	
Overweight	272	929	23.12	26.5		25.52	24.74	
Normal weight	157	784	13.43	21.1		14.73	20.87	
Sleep time (%)					0.4157			0.118
Short sleep time	263	841	20.75	19.21		24.67	22.39	
Normal sleep time	803	2,915	79.25	80.79		75.33	77.61	
Age (%)					< 0.001			< 0.001
Elderly	920	1,102	84.8	27.61		86.3	29.34	
Youth	24	1,237	2.37	33.75		2.25	32.93	
Middle-aged	122	1,417	12.83	38.64		11.44	37.73	

### Associations of sleep duration, age, and covariates with speech hearing threshold

A weighted multivariable regression analysis was performed with sleep duration as the independent variable, speech hearing threshold as the dependent variable, and gender, race, education level, marital status, smoking, blood pressure, cholesterol, diabetes, tinnitus, and BMI as covariates. The results demonstrated: A significant positive association between age and hearing threshold (β = 0.46, *P* < 0.001), indicating that aging substantially elevates hearing thresholds (Table 2); A statistically significant positive association between sleep duration and hearing threshold (β = 0.21, *p*-value = 0.024), suggesting increased sleep duration may adversely affect auditory function (Table 2).

**TABLE 2:** Weighted multifactor regression analysis model of language hearing threshold.

Variables	β	SE	*t*-value	*p*-value	β (95% CI)
Intercept	−8.17	1.19	−6.85	< 0.001	−8.17 (−10.50 ∼−5.83)
**Gender (%)**					
Male					0.00 (reference)
Female	−2.61	0.30	−8.70	< 0.001	−2.61 (−3.20 ∼−2.03)
**Race (%)**					
Non-Hispanic White					0.00 (reference)
Non-Hispanic Black	−3.26	0.41	−8.03	< 0.001	−3.26 (−4.06 ∼−2.47)
Mexican American	−1.19	0.47	−2.52	0.012	−1.19 (−2.11 ∼−0.26)
Other Hispanic	−1.24	0.49	−2.55	0.011	−1.24 (−2.20 ∼−0.29)
Other race-including multi-racial	−0.66	0.47	−1.42	0.155	−0.66 (−1.58 ∼ 0.25)
**Educational (%)**					
High school					0.00 (reference)
Above high school	−1.19	0.36	−3.28	0.001	−1.19 (−1.90 ∼−0.48)
Below high school	2.01	0.45	4.50	< 0.001	2.01 (1.14 ∼ 2.89)
**Marital status (%)**					
Unmarried married					0.00 (reference)
Unmarried married	−1.17	0.30	−3.89	< 0.001	−1.17 (−1.76 ∼−0.58)
**Smoking (%)**					
No					0.00 (reference)
Yes	0.41	0.31	1.35	0.178	0.41 (−0.19 ∼ 1.02)
**Hypertension (%)**					
No					0.00 (reference)
Yes	0.13	0.36	0.37	0.711	0.13 (−0.57 ∼ 0.83)
**High cholesterol (%)**					
No					0.00 (reference)
Yes	−0.71	0.34	−2.09	0.037	−0.71 (−1.37 ∼−0.04)
**Diabetes (%)**					
No					0.00 (reference)
Yes	1.84	0.43	4.28	< 0.001	1.84 (0.99 ∼ 2.68)
**Tinnitus (%)**					
No					0.00 (reference)
Yes	3.33	0.40	8.38	< 0.001	3.33 (2.56 ∼ 4.11)
BMI (kg/m^2^)	0.07	0.02	3.24	0.001	0.07 (0.03 ∼ 0.11)
Age	0.46	0.01	46.21	< 0.001	0.46 (0.44 ∼ 0.48)
Sleep time	0.21	0.09	2.25	0.024	0.21 (0.03 ∼ 0.40)

### Dose–response relationship between sleep duration and speech hearing threshold in the general population

**FIGURE 2:**
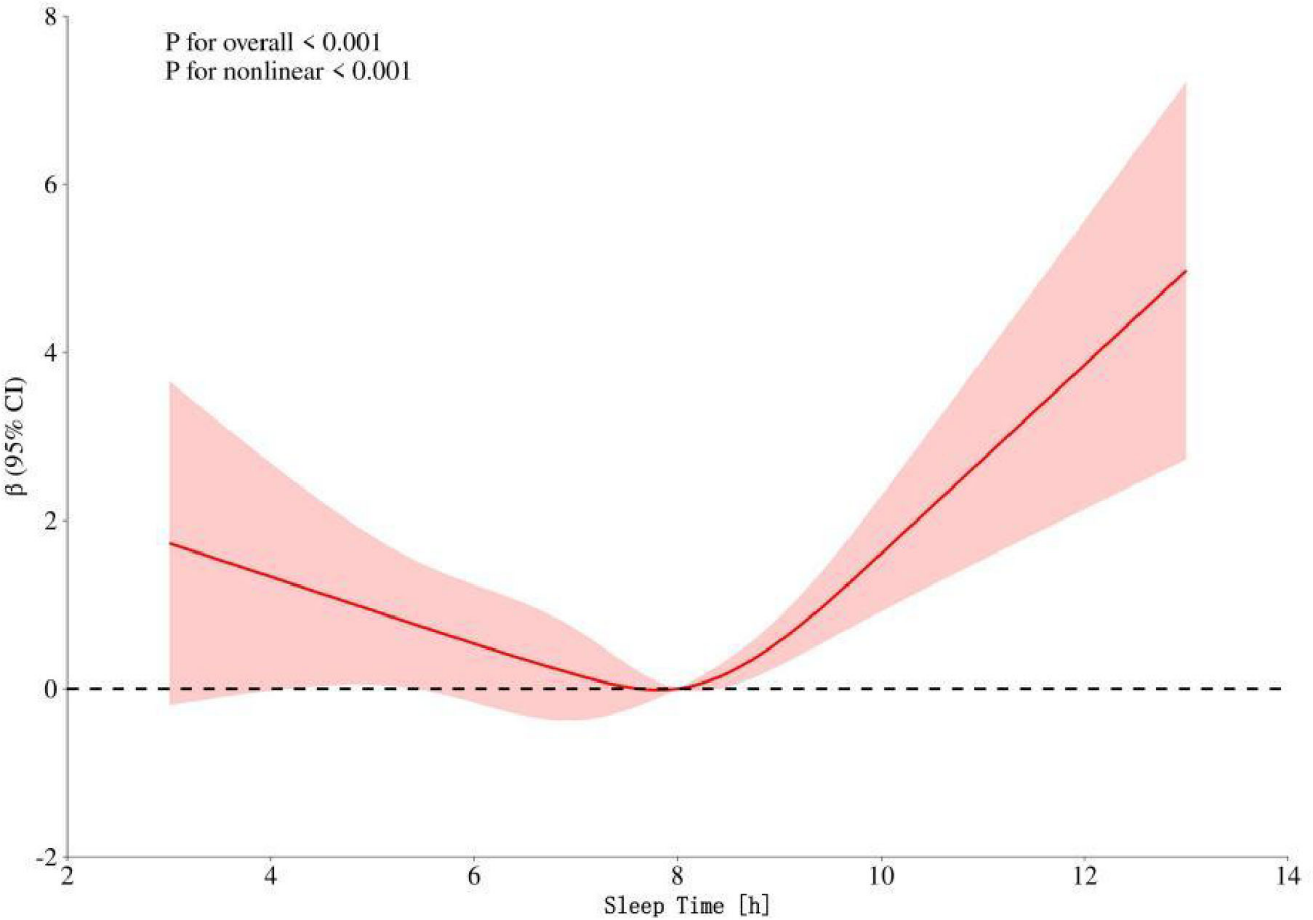
Dose–response relationship between speech frequency and sleep time in the general population. The red shading represents the 95% CI.

Restricted cubic spline regression analysis showed that after adjusting for factors such as gender, race, educational level, marital status, smoking status, blood pressure, cholesterol level, diabetes status, tinnitus status, BMI, and age, the relationship between sleep duration and hearing threshold presented a U–shaped curve pattern (*p*-overall trend < 0.001, *p*-non-linearity < 0.001). The critical turning point occurred when the sleep duration was 8 h. Before this turning point, increasing sleep duration had a protective effect on the hearing threshold; after this turning point, excessive sleep duration led to an increase in the hearing threshold. See Figure 2 for details.

### Dose–response relationship between sleep duration and speech hearing threshold in different gender groups

**FIGURE 3:**
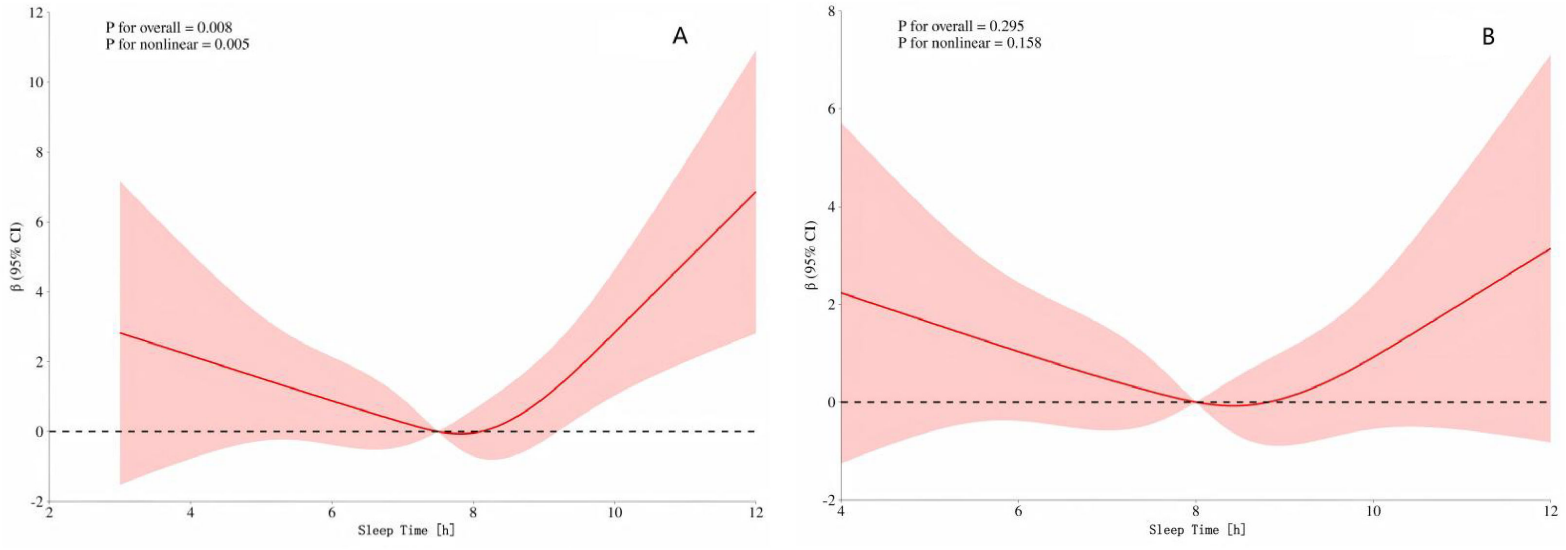
**(A)** Dose–response of speech freq. and sleep time in men **(B)** Dose–response of speech freq. and sleep time in women. The red shading represents the 95% CI.

To further analyze whether there are differences in the dose–response relationship between sleep duration and speech hearing threshold between men and women, we conducted a stratified analysis by gender. The results (Figure 3A) showed that there was a U–shaped curve relationship between sleep duration and speech hearing threshold in men (*p*-overall trend < 0.001, *p*-non-linearity < 0.001). The critical turning points occurred when the sleep duration was 7 and 8 h. Before these two turning points, increasing sleep duration had a protective effect on the speech hearing threshold; after the turning points, excessive sleep duration led to an increase in the speech hearing threshold. However, (Figure 3B) the dose–response relationship between sleep duration and speech hearing threshold in women did not reach a significant level (*p*-overall trend = 0.295, *p*-non-linearity = 0.158).

### The dose–response relationship between sleep duration and speech hearing threshold in different age groups

**FIGURE 4:**
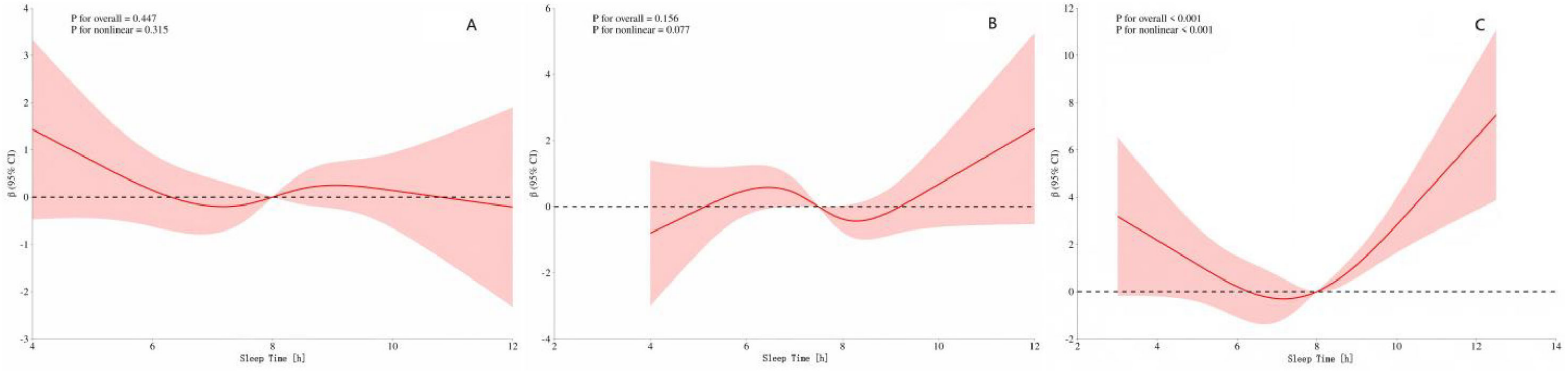
**(A)** Speech frequency-sleep time in youth **(B)** Speech frequency-sleep time in middle-aged **(C)** Speech frequency-sleep time in the elderly. The red shading represents the 95% CI.

To further explore whether there are differences in the dose–response relationship between sleep duration and speech hearing threshold among different age groups, we conducted a stratified analysis by classifying the participants into young, middle-aged, and elderly (aged 60 and above) groups. The results (Figure 4C) showed that there was a U–shaped curve relationship between sleep duration and speech hearing threshold in the elderly group (*p*-overall trend < 0.001, *p*-non-linearity < 0.001). The critical turning points occurred when the sleep duration was 7 and 8 h. Before these two turning points, increasing sleep duration had a protective effect on the speech hearing threshold; after the turning points, excessive sleep duration led to an increase in the speech hearing threshold. However, (Figures 4A, B) the dose–response relationships between sleep duration and speech hearing threshold in the young and middle-aged groups did not reach a significant level (for the young group: *p*-overall trend = 0.447, *p*-non-linearity = 0.315; for the middle-aged group: *p*-overall trend = 0.156, *p*-non-linearity = 0.777).

## Discussion

Hearing loss (HL) is a major global public health issue that severely affects the quality of life of millions of people (Wilson and Tucci, 2021). Although previous studies have confirmed that multiple factors, such as age, gender, occupational noise exposure, etc., are significantly associated with the risk of HL (Gopinath et al., 2021; Reavis et al., 2023; Lin, 2024), the role of sleep duration, as a potentially modifiable risk factor, has not been fully elucidated. Previous research has shown that abnormal sleep (either too short or too long) is associated with various health conditions, including cardiovascular diseases, metabolic syndrome, and cognitive decline (Ma et al., 2020; Tao et al., 2021; Xie et al., 2021). However, the relationship between sleep duration and hearing threshold remains a relatively unexplored area. Based on the large-scale population data from the NHANES in the United States, this study systematically investigated the association between sleep duration and hearing threshold (characterized by the PTA of speech frequencies) among adults aged 20 to 69 years using multiple linear regression and RCS regression analysis.

The key finding of this study is the revelation of a significant U–shaped curve relationship between sleep duration and hearing threshold (*p*-overall trend < 0.001, *p*-non-linearity < 0.001), with the critical turning point at 8 h of sleep. Specifically, before reaching 8 h of sleep, prolonging sleep duration has a protective effect on hearing threshold; however, after exceeding 8 h, the prolongation of sleep duration is associated with an increase in hearing threshold (i.e., hearing decline). The discovery of this non-linear relationship has important clinical and public health implications. Univariate analysis suggested that variables such as gender, age, race, educational level, marital status, smoking history, blood pressure, cholesterol level, diabetes status, tinnitus, and BMI were all significantly associated with the distribution of HL. In the initial multiple linear regression model, after adjusting for the above confounding factors, there was a significant increasing trend in hearing threshold for every one-hour increase in sleep duration (β = 0.21, *p*-value = 0.024). This linear association emphasizes the importance of sleep duration as an independent influencing factor for hearing health, suggesting that clinicians should consider sleep habits when evaluating patients’ hearing problems. Improving sleep patterns may be a potential strategy for preventing or delaying HL. This finding is consistent with existing literature reports. For example, the study by Yévenes-Briones et al. (2023) also observed a significant association between sleep duration and hearing threshold.

However, more in–depth RCS non-linear analysis revealed a more complex dose–response relationship, namely the aforementioned U–shaped curve pattern. Existing biological mechanism studies provide some support for this finding. On the one hand, a large amount of evidence shows that insufficient sleep significantly increases the risk of HL (Wu et al., 2022; Liu et al., 2023). On the other hand, some studies have also suggested that excessive sleep is also associated with hearing impairment. For example, the study by Nakajima et al. (2014) found a positive linear association between HL at 4,000 Hz and sleep duration. Moderate sleep (e.g., 7–8 h) is considered to be beneficial for optimizing blood circulation and metabolic activities, promoting the clearance of waste in the inner ear and tissue repair, thus playing a protective role in hearing (Hill, 1898). In contrast, excessive sleep may have a negative impact on inner ear function through various pathways: Cardiovascular and circulatory effects: Excessive sleep may lead to cardiovascular dysfunction and reduced efficiency of the circulatory system, thereby damaging the microcirculation of the inner ear. The inner ear (especially cochlear hair cells) is highly sensitive to oxygen and nutrient supply and depends on sufficient and efficient blood flow. Studies have shown that prolonged bed rest can reduce vascular patency, blood flow velocity, and affect microvascular function (Gonzales et al., 2022). Inflammation and oxidative stress: Both insufficient and excessive sleep may induce systemic inflammatory responses and elevated oxidative stress levels (Basta et al., 2022; Irwin et al., 2023). This may exacerbate cell damage in the inner ear microenvironment. In particular, excessive sleep is associated with elevated levels of inflammatory markers such as interleukin-6 (IL-6) and C-reactive protein (CRP) (Irwin et al., 2016; Dolsen et al., 2021; Iakunchykova et al., 2024), suggesting that the inflammatory pathway may play an important role in HL related to excessive sleep. Evidence from animal models: Animal experiments further support the deafness-causing mechanism of sleep deprivation. For example, sleep deprivation can lead to an increase in the level of pro-inflammatory cytokine IL-1β in Wistar rats, accompanied by a decrease in the level of distortion product otoacoustic emissions (DPOAE, reflecting the function of outer hair cells in the cochlea), ultimately resulting in HL (Nolan, 2020). This suggests that inflammation-factor-mediated damage to outer hair cells in the cochlea may be a potential pathological mechanism for deafness caused by short-term sleep.

The above mainly elaborates on the non-linear relationship pattern in the general population. However, individual differences (especially gender and age) may significantly affect the specific association pattern between sleep duration and hearing threshold. Gender stratification: This study found that there was a significant U–shaped curve relationship between sleep duration and hearing threshold in men (with critical turning points at 7 and 8 h), while no significant association was observed in women. This difference may be related to inherent physiological and social-behavioral factors between genders. Previous studies have generally observed that men have an earlier onset (Nolan, 2020), higher prevalence (Helzner et al., 2005), and more severe degree (Pearson et al., 1995) of HL, which was previously attributed to differences in noise exposure. However, even after controlling for noise exposure, the gender difference still persists (Pearson et al., 1995), suggesting the role of intrinsic biological factors (such as sex hormones). Both clinical and basic research have shown that estrogen may have a protective effect on hearing (Delhez et al., 2020; Shuster et al., 2021). The significant decline in estrogen and progesterone levels during female menopause is associated with an accelerated process of HL (Kim et al., 2002). Evidence supporting this view includes that post-menopausal women receiving estrogen replacement therapy show a protective effect against HL (Kim et al., 2002). Age stratification: In the elderly population, there was also a significant U–shaped curve pattern between sleep duration and hearing threshold (with critical turning points at 7 and 8 h), while no significant association was found in the young and middle-aged populations. This finding may be related to the unique physiological state and disease burden of the elderly population. Epidemiological studies have shown that high-frequency HL worsens with age, and the progression is faster in men (Wang and Puel, 2020). Although genetic predisposition and age-related cellular changes are the main mechanisms of presbycusis (Paulsen et al., 2019; Naz, 2022; Vaden et al., 2023), multiple intrinsic metabolic and medical factors [such as hypertension (Greenberg et al., 2024), diabetes (Przewoźny et al., 2015), and hypercholesterolemia (Sommer et al., 2017)] also significantly affect the occurrence and development of HL. Elderly people often have multiple chronic diseases, which may damage the microcirculation and metabolic homeostasis of the inner ear, thus weakening the protective effect of moderate sleep on hearing. In addition, lifestyle changes in old age (such as retirement and reduced social activities) may also affect their sleep patterns (duration and quality), thereby indirectly affecting hearing threshold.

### Research advantages and limitations

The primary advantage of this study lies in the utilization of the large-scale and nationally representative NHANES database. Standardized hearing measurements and a validated questionnaire were employed to collect sleep data, and the advanced RCS method was used to explore non-linear relationships. Meanwhile, multiple potential confounding factors were fully adjusted. However, there are several limitations that need to be noted: Cross-sectional design: The cross-sectional design of the study restricts the inference of causal relationships. Abnormal sleep duration may be the cause or result of HL, or both may be influenced by common factors. Assessment of sleep duration: Sleep duration was obtained based on self-reported questionnaires, which may be subject to recall bias or reporting errors. Although the NHANES questionnaire has been validated, the consistency between its data and objective sleep monitoring data (such as polysomnography, PSG) still needs to be carefully considered. Residual confounding: Despite adjusting for multiple covariates, there may still be unmeasured or incompletely controlled confounding factors (such as detailed noise exposure history, specific types of sleep quality/disorders, and the use of certain medications) that can affect the results. Exploration of mechanisms: This study is an observational epidemiological study. The biological mechanisms (Obstructive sleep apnea, inflammation, oxidative stress, and circulatory disorders) discussed are mainly supported by the literature. Future basic experiments and mechanism studies are required to further verify their specific roles in the sleep-hearing relationship.

## Conclusion

According to the analysis results of the NHANES, it was found that the relationship between sleep duration and hearing threshold shows a U–shaped curve pattern, and the critical turning point occurs at a sleep duration of 8 h. Before the turning point, increasing sleep duration has a protective effect on the hearing threshold; after the turning point, excessive sleep duration leads to an increase in the hearing threshold. When stratified by gender and age, the sleep duration and hearing threshold of men and the elderly also show a U–shaped pattern. Short-term and long-term sleep are harmful to the hearing of some American adults. Therefore, preventive strategies need to be implemented to reduce the potential burden associated with HL. In the future, prospective longitudinal studies with an increased sample size are needed, and other analytical methods, such as Mendelian randomization analysis, should be used to improve our research methods. In addition, basic research can help understand the biological mechanism of the relationship between sleep duration and HL.

## Data Availability

The raw data supporting the conclusions of this article will be made available by the authors, without undue reservation.

## Footnotes

1
https://wwwn.cdc.gov/nchs/nhanes/default.aspx

2
https://wwwn.cdc.gov/Nchs/Data/Nhanes/Public/2015/DataFiles/AUX_I.htm
